# Urolithin A as a Potential Agent for Prevention of Age-Related Disease: A Scoping Review

**DOI:** 10.7759/cureus.42550

**Published:** 2023-07-27

**Authors:** Breanne Kothe, Sarah Klein, Stephanie N Petrosky

**Affiliations:** 1 Medical School, Nova Southeastern University Dr. Kiran C. Patel College of Osteopathic Medicine, Fort Lauderdale, USA; 2 Nutrition, Nova Southeastern University Dr. Kiran C. Patel College of Osteopathic Medicine, Fort Lauderdale, USA

**Keywords:** cholangiocarcinoma, colorectal cancer, neoplastic disease, photo-aging, osteoporosis, sarcopenia, neurodegenerative disease, age-related disease, aging, urolithin a

## Abstract

The aging of an organism is hallmarked by systemic loss of functional tissue, resulting in increased fragility and eventual development of age-related neurodegenerative, musculoskeletal, cardiovascular, and neoplastic diseases. Growing scientific evidence points to mitochondrial dysfunction as a key contributor in the aging process and subsequent development of age-related pathologies. Under normal physiologic conditions, the body removes dysfunctional mitochondria via an autophagic process known as mitophagy. Urolithin A (UA), a metabolite produced when gut microflora digests the polyphenol compounds ellagitannin and ellagic acid, is a known inducer of mitophagy via several identified mechanisms of action. The primary objective of this scoping review is to identify and summarize the clinical relevance of UA supplementation in the prevention of age-related pathology and diseases. A computer-assisted literature review was performed using PubMed and EMBASE for primary source research articles examining UA supplementation and aging-related pathologies. A total of 293 articles were initially identified from a database search, and 15 articles remained for inclusion in this review, based on predetermined criteria. Analysis of the 15 identified publications demonstrated that UA holds potential as a dietary intervention for slowing the progression of aging and preventing the development of age-related disease. This review also illustrates the potential role that mitochondrial health and inflammation play in the progression of age-related pathology. Identifying the clinical relevance of UA supplementation in the prevention of age-related pathology and diseases will help further the focus of research on treatments that may improve the longevity and quality of life in patients at risk for these comorbidities.

## Introduction and background

Mitochondrial dysfunction and inflammation in age-related disease 

The aging of an organism is hallmarked by cellular senescence and the accumulation of cellular dysfunction, resulting in the systemic deterioration of tissue [1]. This systemic loss of functional tissue results in increased fragility and eventual development of age-related neurodegenerative, musculoskeletal, cardiovascular, integumentary, and neoplastic diseases [1]. Growing scientific evidence points to mitochondrial dysfunction as a key contributor in the aging process and the subsequent development of age-related disease [1]. Senescent tissues, characteristically seen in aging, display an array of structural and functional changes within mitochondria [1]. Among these observed changes, the accumulation of mitochondrial deoxyribonucleic acid (DNA) mutations and disruptions of oxidative phosphorylation are of particular significance [1]. Furthermore, mitochondrial dysfunction can both cause and result from increased oxidative stress [2]. The mitochondria house a complex collection of energy-producing reactions within the cell, which make up the process known as mitochondrial respiration [2]. During these cellular energy-producing reactions, the production of chemical agents known as reactive oxygen species (ROS) also occurs [2]. ROS can cause damage to cellular structures, and thus, an excess of ROS can wreak havoc on the cell [2]. When a cell becomes senescent, the mitochondrial membrane potential is altered, leading to the loss of the proton gradient [1]. This results in an imbalance of energy supply and demand, as well as increased formation of ROS [1]. These elevated levels of ROS lead to local tissue damage via inflammatory pathways and contribute to neuronal necrosis seen in neurodegenerative disease [3], as well as the accumulation of abnormal mitochondria seen in sarcopenia [4]. Moreover, increased ROS can cause further oxidative damage to the mitochondria, resulting in the downregulation of mitochondrial gene expression and further hindrance of oxidative phosphorylation [3].

Mitophagy and urolithin A 

Under normal physiologic conditions, the body removes dysfunctional mitochondria via an autophagic process called mitophagy [5]. Impaired mitophagy can lead to the accumulation of dysfunctional mitochondria, contributing to the development of age-related pathology [5]. Current research aims to identify potential interventions that promote mitochondrial health and induction of mitophagy in aging tissues [6]. Urolithin A (UA), a metabolite produced when gut microflora digests the polyphenol compounds ellagitannin and ellagic acid, is a known inducer of mitophagy via several identified mechanisms of action [7]. Researchers have identified UA as a potent activator of the phosphatase and tensin homolog (PTEN)-induced kinase-1 (PINK1)/parkin-dependent mitophagy pathway, which leads to the selective ubiquitination of mitochondrial proteins for phagolysosomal clearance [7]. UA has also demonstrated activation of several PINK1/parkin-independent pathways, ultimately resulting in the degradation of defective mitochondria [7].

Potential implications for UA in age-related disease

Nutritional intervention in various diseases and ailments has gained traction in recent years due to its minimally invasive nature, affordability, and wide accessibility. UA supplementation stands among these interventions as a potential protective agent against the development of age-related disease [7]. UA has a broad and varied reach in terms of potential implications as a preventive dietary supplement for the aforementioned diseases. Dietary consumption of UA has been found to induce both autophagy and apoptosis, suggesting the ability to inhibit human colorectal cancer cell metastasis [8]. UA has also been found to selectively promote cellular senescence, an anticancer mechanism that prevents irreversible cell cycle progression, in colorectal cancer cell lines, suggesting that regular consumption of ellagitannin-rich food may be an effective chemopreventive strategy against colorectal cancer [9]. There is also evidence that complications of type II diabetes mellitus may involve overactivation of a protein kinase pathway, known as the Ak-strain transforming (Akt) kinase pathway, which plays a role in mediating intracellular effects of insulin [10]. UA is a statistically significant inhibitor of the phosphorylation of Akt and, therefore, may have beneficial effects on diabetes-induced cardiovascular complications [10]. Research has also found that UA treatment markedly induces mitophagy in nucleus pulposus cells, suggesting that UA could slow the progression of intervertebral disc degeneration, a common cause of back pain in older adults [11]. Research shows that UA significantly increased type I collagen expression, reduced matrix metalloproteinase-1 expression, and reduced intracellular ROS in senescent human skin fibroblasts, suggesting UA’s potential as an anti-aging supplement [12]. Furthermore, the concentrations of UA that triggered autophagy were consistent with those found in the intestine, which suggests that dietary polyphenols play an important role in body functions [8]. These polyphenol compounds that UA is derived from are abundant in various berries, nuts, pomegranates, spices, and other various food options and are therefore widely accessible to large populations, further supporting its use as a natural remedy in aging and age-related disease provoked by mitochondrial dysfunction [7,10].

Objectives 

Age-related pathology and diseases, such as Alzheimer’s disease and various musculoskeletal, cardiovascular, and neoplastic diseases, remain as the major causes of mortality and reduced quality of life and currently have no cures. Additionally, current antineoplastic drug regimens are not always efficacious and come with many unwanted side effects. Given the progressive interest in natural remedies and health-conscious practices in disease prevention, further analysis of current literature that assesses UA supplementation in human test subjects or human cell lines was warranted to provide practicing clinicians with organized evidence for utilizing UA within their patient populations. In summary, identifying the clinical relevance of UA supplementation in preventing aging and developing age-related disease will help further focus research on treatments that may improve longevity and quality of life in patients at risk for age-related pathology.

## Review

Search strategy 

A computer-assisted scoping review was performed using PubMed and EMBASE for primary source research articles examining UA supplementation in the prevention or reversal of aging and age-related disease, such as neurodegenerative, musculoskeletal, and cardiovascular disease. The database key search terms included “Urolithin A” and “aging” or “age related disease” or “mitophagy” or “autophagy” or “sarcopenia” or “cardiovascular disease” or “joint degeneration” or “neurodegenerative disease.” Articles were filtered to include those published after 2017 to improve the efficacy of the review and capture the most recent and prominent research. The reference list of retrieved articles was also considered when found to be relevant and if those additional articles fit the search criteria but were not discovered through the initial search. The relevance of these articles was assessed through a hierarchical approach, first with an evaluation of the title, then the abstract, followed by the full manuscript. For any articles not freely available, access was gained through the Nova Southeastern University library when available.

Selection criteria 

**Figure 1:**
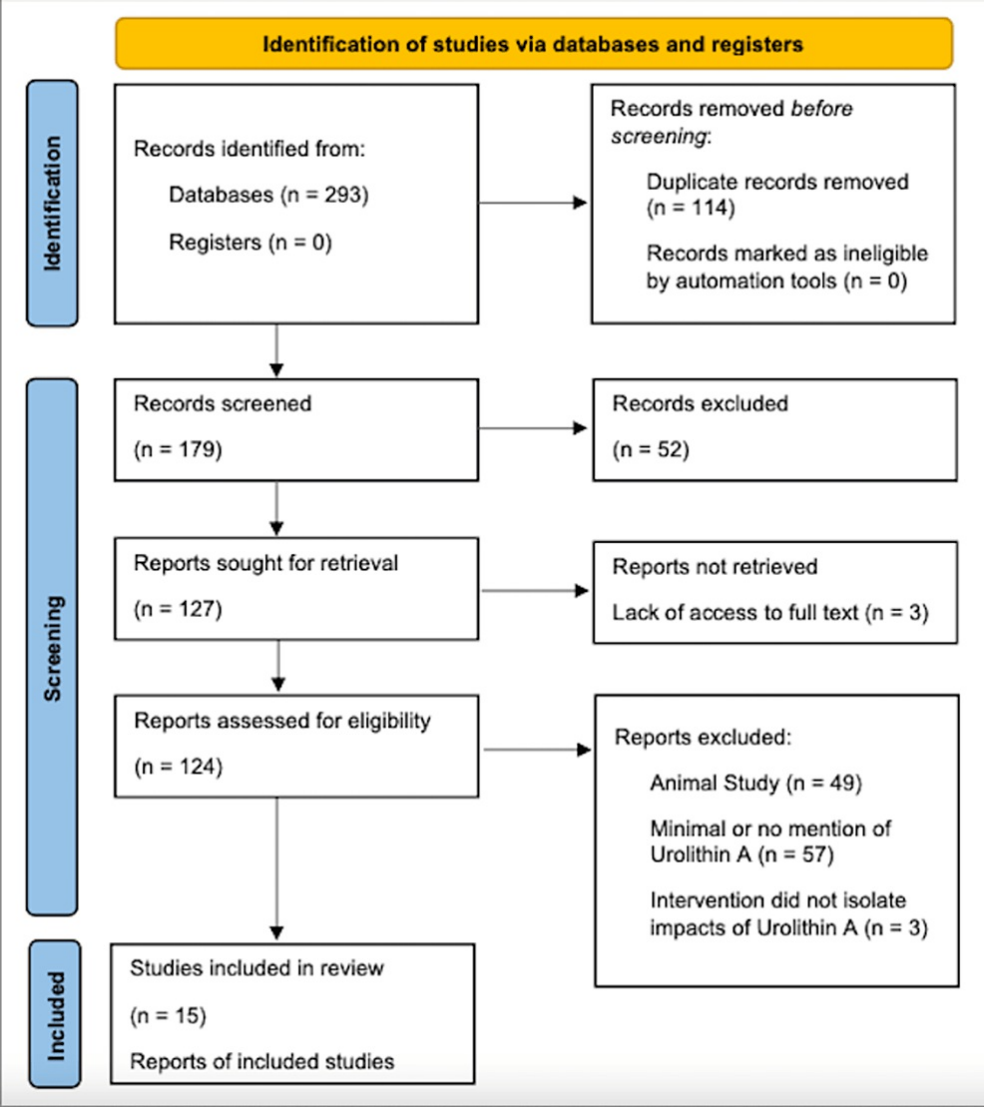
Selection process flow diagram PRISMA flowchart [8]

Studies were eligible if they examined UA supplementation in age-related processes or diseases and provided clinically relevant intervention measures. During the article screening process, the writers determined that studies investigating the impact of UA on neoplastic disease were also relevant to the objectives of this review. The decision was made to include articles investigating neoplastic disease as an age-related pathology even though it was not explicitly mentioned in the key search terms. Eligible study designs included randomized control trials (RCT), prospective and retrospective studies, cross-sectional studies, case studies, and other studies on human subjects and/or human cell lines. Exclusion criteria included meta-analyses, systematic reviews, editorials, non-human studies, and limited access to the available text. Due to the novel nature of the investigation of this topic, specific study populations were not considered as a factor in determining inclusion eligibility for this review. Additionally, because studies completed in animal models only provide theoretical data regarding the safety and efficacy of a substance, these studies cannot confirm these qualities within humans and thus were omitted from this review. A flow diagram (Figure 1) was developed using the Preferred Reporting Items for Systematic Reviews and Meta-Analyses (PRISMA) 2020 outline [13].

Results

A total of 293 articles were identified from the initial electronic database search. Fifty-two articles were excluded based on lack of relevance found during the title and abstract screening, and 114 duplicates were removed. Of the remaining 179 articles, 109 were excluded for containing minimal or no mention of UA, focusing on an intervention that utilized UA in tandem with another agent, or testing in animal models. Three articles were excluded due to a lack of access to the full text. A total of 15 articles remained for inclusion in this review (Table 1). All of the identified publications evaluated UA’s impacts on aging and age-related pathology. Included studies largely investigated the impact of UA in first world populations, though details on specific demographics for the investigated populations were not always explicitly expressed. For publications that included human cell line studies in addition to animal model studies, findings from the animal model portion of the study were excluded from this review.

**Table 1:** Summary of pertinent findings HCL, human cell line; UA, urolithin A; MMP, matrix metalloproteinase; ATP, adenosine triphosphate; ROS, reactive oxygen species; SIRT1, sirtuin 1; RCT, randomized controlled trial; PO, per os; CRP, C-reactive protein; RANKL, receptor activator of nuclear factor 𝜅B ligand; mRNA, messenger ribonucleic acid; PARK2, Parkinson’s disease-associated gene 2; SQSTM1, sequestosome 1; MMP-1, matrix metalloproteinase 1; DNA, deoxyribonucleic acid; UVA, ultraviolet A; Akt, Ak strain transforming kinase; MMP-9, matrix metalloproteinase 9; CCA, cholangiocarcinoma

-	Author	Study type	Intervention	Sample size	Pertinent results
1	Esselun et al. (2021) [14]	HCL	1 or 10 μM UA in vitro	N/A	10 μM doses of UA decreased MMP and ATP levels; 1 uM dose of UA increased mitochondrial gene expression for biogenesis and oxidative phosphorylation
2	Kim et al. (2020) [15]	HCL	1.25, 2.5, or 5 μM UA in vitro	N/A	Pretreatment with UA significantly increased neuronal cell viability in a H_2_O_2_-induced oxidative damage model; pretreatment with UA decreased ROS in neuronal cells in a H_2_O_2_-induced oxidative damage model; pretreatment with UA decreased concentrations of pro-apoptotic proteins in a H_2_O_2_-induced oxidative damage model
3	Lee et al. (2021) [16]	HCL	100 nM UA in vitro	N/A	UA treatment decreased high glucose-induced ROS; UA treatment inhibits high glucose-stimulated mitochondrial calcium accumulation
4	Velagapudi et al. (2019) [17]	HCL	2.5-10 μM in vitro	N/A	UA treatment reversed the effects of autophagy inhibitors in human neural cells; UA treatment increased SIRT-1 and autophagic activity in human neural cells
5	Andreux et al. (2019) [18]	RCT	250, 500, 1,000, or 2,000 mg UA PO	n = 60	Oral doses up to 1,000 mg of UA are both safe and tolerable; UA is bioavailable at doses ranging from 250 to 1,000 mg in elderly adults; 500-1,000 mg doses for 28 days showed improved mitochondrial biomarkers
6	Liu et al. (2022) [19]	RCT	1,000 mg UA PO	n = 66	Oral doses of 1,000 mg resulted in significant improvement in muscle endurance; plasma levels of acylcarnitines, ceramides, and CRP decreased with UA supplementation
7	Singh et al. (2022) [20]	RCT	500 or 1,000 mg UA PO	n = 88	UA supplementation showed significant improvement in muscle strength, aerobic endurance, and physical performance; decreased plasma acylcarnitines and CRP; and increased expression of proteins associated with mitophagy
8	Tao et al. (2022) [21]	HCL	5 or 10 μM UA in vitro	N/A	UA treatment inhibited RANKL-induced osteoclastogenesis in bone marrow macrophages; UA treatment significantly reduced bone resorption and osteoclastic gene expression; UA treatment significantly improved autophagic abilities of bone marrow macrophages
9	D’Amico et al. (2022) [22]	HCL	6.25 or 12 μM UA in vitro	N/A	UA treatment significantly improved cellular respiration in primary human chondrocytes; 24-hour UA treatment did not increase mRNA expression of mitochondrial biogenesis or oxidative phosphorylation genes; UA treatment significantly upregulated expression of PARK2 and SQSTM1
10	Liu et al. (2019) [12]	HCL	50 μM in vitro	N/A	UA treatment significantly increased type I collagen expression and reduced MMP-1 expression; UA treatment inhibited cell proliferation due to cell cycle arrest
11	Liu et al. (2022) [23]	HCL	0.1 or 1 μM in vitro	N/A	Treatment with UA resulted in the prevention of senescence, DNA damage, and morphologic changes caused by UVA radiation in human dermal fibroblasts; treatment with UA reduced mitochondrial damage in photoaging models
12	Dirimanov and Högger (2019) [10]	HCL	10 μM in vitro	N/A	UA caused statistically significant inhibition of Akt phosphorylation; of the ellagic acid metabolites, UA is the only one that inhibited Akt phosphorylation
13	Zhao et al. (2018) [8]	HCL	1.5, 15, or 30 μM in vitro	N/A	UA treatment dose-dependently decreased cancer cell proliferation, delayed cell migration, and decreased MMP-9 activity; UA treatment induced autophagy and apoptosis in cancer cells; UA treatment promotes cell cycle arrest and inhibited DNA synthesis in cancer cells
14	Giménez-Bastida et al. (2020) [9]	HCL	0.5, 1, or 10 μM in vitro	N/A	Treatment with UA led to a dose-dependent decrease in cancer cell proliferation
15	Sahashi et al. (2022) [24]	HCL	10 or 40 μM UA in vitro	N/A	UA treatment of 40 μM for 48 hours significantly reduced cell proliferation, resulting in cell cycle arrest in the G2/M phase in HuCCT-1 and SSP-25 cells; UA treatment of 40 μM significantly reduced CCA tumor cell migration and invasion; UA treatment of 10 or 40 μM for 24 hours did not increase apoptosis when compared to controls; 24-hour UA treatment increased autophagy within HuCCT-1 and SSP-25 cells

Discussion

From the analyzed publications, five main age-related disease pathologies have been investigated in studies conducted in humans or human cell lines: neurodegenerative disease [14-17]; musculoskeletal-related disease, including joint degeneration, osteoporotic disease, and sarcopenia [18-22]; cardiovascular disease [10]; integumentary disease [12,23]; and cancer [8,9,24].

Neurodegenerative disease 

Four of the identified articles focused on neurodegenerative disease [14-17]. The primary focus of three of these studies was determining the neuroprotective capabilities of UA in the pathogenesis of Alzheimer’s disease [14,15,17]. In general, pretreatment of various human neuronal cell lines with UA solutions in vitro of concentrations varying from 1 to 10 μM resulted in decreased neuroinflammation and local ROS levels [14-17]. In one study, Velagaputi et al. reported a statistically significant (p < 0.01) and dose-dependent reduction in the inflammatory markers’ tumor necrosis factor alpha (TNF-α) and nitrite with UA pretreatment with doses between 2.5 and 10 μM [17]. Interleukin-6 levels were also decreased in these models; however, the decrease was not found to be significant [17]. Further, 2.5 μM doses of UA resulted in increased nuclear SIRT-1 protein expression, resulting in decreased NF-kB pathway activation and overall decreased production of inflammatory cytokines, in comparison to controls [17]. UA pretreatment was also found to attenuate oxidative stress-induced apoptosis via inhibition of the mitochondrial-related apoptosis pathway [15] and demonstrate a dose-dependent neuroprotective effect in amyloid beta-induced (A𝛽) neurotoxicity models [17].

The fourth neurodegenerative disease-based study in this review examined high glucose-induced neurodegeneration similar to what is seen in advanced diabetes mellitus [16]. UA pretreatment was able to alleviate high glucose-induced mitochondrial ROS accumulation by reducing the mitochondrial influx of calcium generated by the hyperglycemic environment [16]. Moreover, expression of A𝛽-producing enzymes typically seen in hyperglycemia-induced neurodegenerative changes, such as amyloid precursor protein (APP), decreased with UA pretreatment, further suggesting UA’s neuroprotective role [16].

Musculoskeletal-related disease 

Three randomized controlled trials investigating the utility of UA as a preventive agent in the development of sarcopenia were examined in this review [18-20]. Primary test subjects for all three of these studies were untrained middle age and older adults [18-20]. UA doses from 250 mg up to 1,000 mg daily were shown to be readily bioavailable, safe, and tolerable, with no severe adverse effects reported in these populations [18]. Generally, improvements in exercise performance, metabolic profiles, and mitochondrial biomarkers were not observed with supplementation of UA at doses lower than 500 mg per day [18-20]. However, supplementation of UA at 1,000 mg daily for 120 days showed significant improvement in six-minute walk test results [19]. Gait speed and measurements of muscle endurance, such as peak VO2 and estimated VO2 max, also improved over the 120-day treatment period, though this improvement was not statistically significant [20]. Further, UA supplementation with 500 mg daily contributed to reduced serum levels of acylcarnitines, indicating improved fatty acid oxidation and better utilization of fat as an energy source for the body [18-20]. This same dose also showed reduced serum inflammatory markers, such as TNF-α, C-reactive protein, interferon-𝛾, and interleukin-1B, and improved glucose mobilization, indicating enhanced muscle metabolism [18,20]. On the cellular level, improvements in mitochondrial biomarkers were noted as early as 28 days after initiating UA supplementation [18]. Additionally, mitochondrial gene expression was notably upregulated, and Singh et al. observed increased activation of the PINK1/parkin-mediated mitophagy pathway in test subjects, supporting improved mitochondrial health with UA supplementation [20].

The final three musculoskeletal-related studies focus on age-related bone and joint pathology [21,22]. UA supplementation has also shown promise as an anti-aging remedy in the development of osteoporosis [21]. In a 2022 study completed by Tao et al., a significant reduction in bone resorption and osteoclast-related gene expression was seen in human cell samples treated with 5 and 10 μM doses of UA [21]. Treatment with UA also enhanced autophagic activity in bone marrow macrophages, and this increase was maintained after stimulation of increased osteoclastogenesis via the receptor activator of the nuclear factor 𝜅B ligand (RANKL) pathway [21]. Current evidence also suggests that osteoarthritic disease progression may be altered by UA supplementation [22]. Both healthy and osteoarthritic primary human chondrocytes showed improved mitophagy and mitochondrial respiration when treated with UA solution in vitro, when compared to controls in a study by D’Amico et al. [22].

Cardiovascular diseases

Though the impacts of UA in cardiovascular diseases have been largely studied in animal models, there is limited research conducted in humans or using human cell lines on the effects of UA in cardiovascular diseases. However, one study by Dirimanov and Högger looked at the impact of UA on the Akt kinase pathway in human cell lines [10]. This pathway, which has been identified as overactive in diabetes mellitus, is closely related to the cardiovascular complications of this metabolic dysfunction [10]. UA has been shown to significantly inhibit phosphorylation of Akt kinase, potentially leading to reduced atherosclerotic complications in patients with diabetes mellitus [10].

Integumentary disease

One of the most visible signs of aging is the impact that cellular senescence and subsequent degradation of cellular components have on the appearance of our skin. Two studies included in this review investigated the effects of UA on the aging of the skin [12,23]. Human skin fibroblasts treated with UA showed significant increases in type I collagen expression [12]. This type of collagen, which is an important structural component of various tissues, including the skin, contributes to the firmness that many deem youthful [12]. Liu et al. presented evidence suggesting that UA has anti-aging effects on the skin through this mechanism [12]. 

Another study investigated the protective effects of UA against UVA-induced photoaging [23]. This study found that treatment of skin fibroblasts with 0.2 μM doses of UA resulted in decreased extracellular matrix breakdown via reduced activation of matrix metalloproteinases [23]. Additionally, UA treatment prevented various morphologic changes typically observed after UVA exposure and reduced DNA damage, suggesting UA may serve as antineoplastic in this setting as well [23]. The investigators also found that UA treatment prevented skin fibroblast senescence and reduced oxidative stress via increased expression of a transcription factor known as nuclear factor erythroid 2-related factor (NFR2), further demonstrating the anti-aging impacts of UA [23]. 

Neoplastic disease

A few studies focused on the antineoplastic impacts of UA supplementation [8,9,24]. Zhao et al. investigated the impacts of UA in SW620 colorectal cancer cells [8]. In this study, the investigators determined that UA treatment resulted in decreased activity of matrix metalloproteinase-9 (MMP-9), an enzyme responsible for basement membrane and extracellular matrix degradation seen in local invasion of cancer cells [8]. This suggests that UA decreases cancer cell proliferation, delays cell migration, and potentially prevents metastasis in a dose-dependent manner [8]. Giménez-Bastida et al. [9], who also studied UA treatment of human colorectal cancer cells, found that UA treatment was most effective in reducing clonogenic growth when compared to other dietary urolithins, a finding consistent with the work of Zhao et al. [8]. Interestingly, Zhao et al. further identified the appearance of vacuoles containing organelles and cellular fragments, indicating the formation of autolysosomes, which supports UA’s role in increased autophagy [8]. Zhao et al. also demonstrated evidence of increased apoptosis in cancer cells treated with micromolar concentrations of UA, further illustrating the antineoplastic effects of UA [8].

The final study analyzed in this review was a human cell line study completed by Sahashi et al. [24]. This study utilized HuCCT-1 and SSP-25 cells to model cholangiocarcinoma (CCA) in vitro and investigated UA treatment’s impacts in the neoplastic setting [24]. Treatment with 40 μM of UA for 48 hours significantly reduced cell proliferation within tumor cell models and resulted in cell cycle arrest in the G2/M phase [24]. This study further demonstrated a significant reduction of cell migration and invasion with similar doses [24]. Sahashi et al. also found UA treatment to upregulate autophagy within HuCCT-1 and SSP-25 cells. However, no significant increase in apoptosis was identified when compared with controls [24]. Cell cycle arrest, inhibition of cell migration, and invasion and autophagy are all mechanisms that many current cancer treatments target, providing further evidence that UA may play a preventative role in the development and progression of neoplastic disease [24].

Final considerations

Though the publications examined in this review largely lean in support of the use of UA as a potential preventive measure in the mitigation of aging and age-related disease, the strength of this study is limited by the scarcity of published human trials. No studies concluded that UA had a negative effect on any of the studied populations. Three of the 15 studies included in this review were randomized controlled trials measuring UA’s impact on sarcopenia in humans. Thus, current evidence supporting the supplement’s use is strongest for this application. However, more longitudinal studies and human trials across all applications are needed to further explore the utility of UA as a dietary intervention in slowing and preventing the development of various age-related pathologies.

## Conclusions

Analysis of the 15 identified current publications demonstrates that UA holds strong potential as a dietary intervention for slowing the progression of aging and preventing the development of age-related diseases. None of these studies found UA to exhibit overall negative effects on the studied populations, further supporting its potential as an impactful dietary intervention in at-risk populations. This review also illustrates the potential role that mitochondrial health and inflammation play in the progression of age-related pathology. Further, this review suggests that the utilization of UA may offer benefits to patients actively combating the development of sarcopenia. More longitudinal studies and further analysis measuring the efficacy of UA in human trials would provide more robust suggestions on potential nutritional and dietary interventions for patients concerned with age-related pathology.
